# Minding the gap: access to palliative care and the homeless

**DOI:** 10.1186/s12904-015-0059-2

**Published:** 2015-11-18

**Authors:** Lise Huynh, Blair Henry, Naheed Dosani

**Affiliations:** Department of Palliative Medicine, Sunnybrook Health Sciences Centre, Toronto, ON Canada; Division of Palliative Care, Department of Family and Community Medicine, University of Toronto, Toronto, ON Canada; Ethics Centre, Sunnybrook Health Sciences Centre, Toronto, ON Canada; Inner City Health Associates, Toronto, ON Canada; Department of Family & Community Medicine St Michael’s Hospital, Toronto, ON Canada; Division of Palliative Care, Faculty of Health Sciences, McMaster University, Hamilton, ON Canada; Division of Palliative Care, William Osler Health System, Brampton, ON Canada

**Keywords:** Palliative care, End-of-life care, Social determinants of health, Homelessness, Access, Socio-economic status

## Abstract

**Background:**

With an ever increasing number of individuals living with chronic and terminal illnesses, palliative care as an emerging field is poised for unprecedented expansion. Today’s rising recognition of its key role in patients’ illnesses has led to increased interest in access to palliative care. It is known that homelessness as a social determinant of health has been associated with decreased access to health resources in spite of poorer health outcomes and some would argue, higher need. This article aims to discuss the current state of affairs with regards to accessing palliative care for the homeless in Canada.

**Discussion:**

Recent review of the literature reveals differential access to palliative care services and outcomes with differing socio-economic status (SES). Notably, individuals of lower SES and in particular, those who are homeless have poorer health outcomes in addition to poor access to quality palliative care. Current palliative care services are ill equipped to care for this vulnerable population and most programs are built upon an infrastructure that is prohibitive for the homeless to access its services. A preliminary review of existing Canadian programs in place to address this gap in access identified a paucity of sporadic palliative care programs across the country with a focus on homeless and vulnerably-housed individuals. It is apparent that there is no unified national strategy to address this gap in access.

**Summary:**

The changing landscape of the Canadian population calls for an expansion of palliative care as a field and as many have put it, as a right. The right to access quality palliative and end of life care should not be confined to particular population groups. This article calls for the development of a unified national strategy to address this glaring gap in our healthcare provision and advocates for attention to and adoption of policy and processes that would support the homeless populations’ right to quality palliative care.

## Background

With improving medical advances and the predicted increase in life expectancy of many individuals living with chronic and terminal illnesses, palliative care as an emerging field is poised for unprecedented expansion. This recognized need for expansion has led to the evaluation of palliative care as a key community health resource, particularly with recent clear evidence suggesting that early palliative care involvement can even lead to an improvement of both quality of life, and, at times, its longevity [1, 2].

With mounting evidence of such benefits for early access to palliative care, policy attention is slowly being focused on the issue of existing disparities pertaining to access for this important service. This clarion call for better access to palliative care has received additional endorsement based on the recent use of rights language to describe the need for palliative care globally. This concept of palliative care as a right was first proposed by Brennen in 2007 and more recently proclaimed in the 2014 WHO Global Atlas of Palliative Care at End of Life [3, 4].

Unfortunately, in spite of calls for improved resources, few advances have been made with the translation of access of these resources to vulnerable populations even in the developed world, particularly those living in the shadows of society: the homeless. Not surprisingly a review of the palliative care literature reveals that individuals with poor socio-economic status (SES) continue to have poorer access to palliative care services with subsequently poorer outcomes [5–11]. Why does being marginalized by socio-economic standards continue to beget such poor outcomes; particularly in countries with universal health care? While not intended to be a systematic review, this article attempts to summarize the existing literature exemplifying the current inequality in palliative care services, present a structural rationale to explain the current state of affairs, and advance a call to action on this issue.

## Discussion

### The Issue – The homeless: socio-economically disadvantaged and lacking access to healthcare services, including Palliative Care

Poverty and homelessness are growing socio-economic and ultimately political issues in Canada. According to Statistics Canada, approximately 13 % of the Canadian population earns a ‘low income’ and it is estimated that annually, 150 000–300 000 individuals in Canada experience homelessness per year [12, 13]. The problem of homelessness is much more than just a socio-economic or political one as it directly affects health and access to healthcare for an already marginalized subset of our population.

Individuals with a low SES are known to have poorer treatment access and health outcomes and these consequences are well exemplified in the homeless. Compared to the general population, those who are homeless have higher morbidity and mortality rates with an average life expectancy of 42–52 years (compared with 79–83 years for the general population), an increased prevalence of hepatitis of 29 times, heart disease by 5 times, cancer by 4 times, and diabetes by 2 times [5]. From a cancer point of view, a retrospective cohort study from a US national cancer registry 2008 revealed that low SES was associated with a more advanced disease stage and less aggressive treatment for individuals with breast, prostate, and colon cancer. This same study found a higher mortality rate for individuals of low SES with breast and prostate cancer [6]. Several barriers related to access to care have been identified and include: negative past experiences with healthcare workers, a lack of trust with healthcare workers, and a lack of comfort with systems not employing harm reduction strategies [5].

In addition to worse treatment access and health outcomes, individuals of low SES have been found to also have less access to community palliative care resources and hospice care. A literature review by Walshe et al. revealed that individuals who are older, male, from an ethnic minority, single, without a home caregiver, and who are socioeconomically disadvantaged (including lower education, not owning a home) are less likely to access community palliative care services [7]. A more recent review by Lewis et al. yielded similar conclusions [8]. These reviews primarily included literature from the UK, Canada, US, and Australia and pertain to patients with both malignant and non-malignant diagnoses.

In the UK a recent retrospective cohort study revealed that referral rates to hospice and home care are significantly lower in regions of lower SES in defiance of a higher cancer incidence and mortality rate [9]. A Canadian study by Daneault et al. found that HIV positive individuals of low SES had poorer access to a multidisciplinary palliative care healthcare team and were less likely to die at home [10]. Similarly, a recent US study found that, individuals of a lower SES were less likely to receive continuous care and to achieve a home death, with higher rates of transfer to institutions near the end of life [11].

In spite of known poorer health status and outcomes which would warrant comprehensive management through quality palliative care, it is clear from the emerging literature that individuals from a low SES do not have access to this care.

### How are Canadian organizations addressing the issue?

Notwithstanding abundant literature showing palliative care access and need discrepancy, it appears that little effort has been made to mend this gap in healthcare access. This is evidenced by the paucity of literature on programs with aims to improve palliative care service delivery and outcomes to those of low SES and in particular, to the homeless. In Canada, though considerable efforts have been done in the primary care setting to identify poverty and poor SES as determinants of health, little such efforts exist in the palliative care community.

The scarce available literature related to this issue points to isolated and independent efforts existing across the country, primarily located in major urban centres. Examples of such efforts include the establishment of a social determinant of health screening tool at the Toronto Sick Kids hospital for palliative care patients, a hospice aimed to provide care to HIV patients in Toronto and in Calgary, and shelter based palliative care for the terminally ill homeless in Ottawa, Toronto and Vancouver [14–17]. More recently, an ambulatory palliative care group was established in Toronto to provide palliative care to terminally ill homeless individuals in mobile and shelter settings.

Given the independent nature of the aforementioned initiatives, very little has been published by way of program evaluation and feasibility. The literature available however, suggests that palliative care programs for homeless individuals are effective in helping the homeless die comfortably and with dignity, are appreciated by the homeless population, and are cost-effective with an estimated health-care cost saving of over $1.3 Million in an Ottawa evaluative study published in 2006 [16].

In spite of the current clear need for improved palliative care delivery for Canadian populations of low SES and the successes of present individual initiatives, there continues to be no national unified strategy for providing palliative care to this group. Current systems-level palliative care campaigns including the national Speak Up campaign on advance care planning and the Canadian Hospice and Palliative Care Association’s Quality End of Life Care Coalition place emphasis on home-based care as well as resources and support for family members. Though helpful for the typical Canadian population, this projected model will not address the needs of the homeless who are often without a home and estranged from family and friends. Indeed, a current Canadian qualitative study echoed that these intrinsic aspects of current end-of-life care programs are barriers to allowing the homeless access quality palliative care [17]. The review moreover demonstrated that operational policies of many palliative care programs (e.g. anti-drug, conduct and behavioural codes) inherently prohibit the homeless and most vulnerable from being included [17]. To address this issue, McNeil et al. have called for the creation of initiatives that cater to the needs of the socio-economically disadvantaged by adopting flexible, low-threshold strategies. Participants from their recent qualitative study point out the importance of adopting a harm reduction strategy to foster trust with the homeless population by demonstrating a commitment to serve them and an awareness of their life circumbstances [17]. In addition, partnering with local community agencies, and strengthening the training on the complex end-of-life care issues related to the dying homeless emerged as additional strategies to foster trust with the homeless population and thereby increase their access to end of life care services [17].

## Summary and conclusion

The history of palliative care is one of a movement that grew from an initial call to advocate on behalf of dying patients whose needs were not being met by the conventional medical system of its day. It was a response to the abandonment patients and families experienced and a recognized need for dignity to be infused into this most sacred and final act of our existence. This value was and remains its core mission.

However, palliative care has become more recognized by mainstream medicine and perhaps more medicalized as a result of that encounter. In truth, our current palliative care delivery system requires a degree of monetary, in-person, and infrastructure support that often creates barriers for the homeless population; barriers they simply cannot overcome. This reality affects access to quality palliative care, not only at the end of life but also along the trajectory of a disease- where recent evidence supports its use.

Current palliative care services are glaringly unequipped to properly meet the needs of the homeless. McNeil et al. have called for the creation of initiatives that cater to the needs of the poor by adopting flexible, low-threshold strategies, partnering with local community agencies, and by strengthening the training on end-of-life care issues amongst those who care for the homeless population [17]. If good quality end-of-life care is a right, it is one for all members of society and we need to re-envision our services to better support those who remain vulnerable.

## Abbreviations

HIV: human immunodeficiency virus
SES: socio-economic status
